# Effect of BRCA1 on epidermal growth factor receptor in ovarian cancer

**DOI:** 10.1186/1756-9966-32-102

**Published:** 2013-12-09

**Authors:** Da Li, Fang-Fang Bi, Ji-Min Cao, Chen Cao, Chun-Yan Li, Qing Yang

**Affiliations:** 1 Department of Obstetrics and Gynecology, Shengjing Hospital, China Medical University, No. 36, Sanhao Street, Heping District, Shenyang 110004, China; 2 Department of Physiology and Pathophysiology, Institute of Basic Medical Sciences, Chinese Academy of Medical Sciences, School of Basic Medicine Peking Union Medical College, Beijing 100005, China; 3 Department of Pathology, Chinese PLA General Hospital, Beijing 100853, China; 4 Department of Histology and Embryology, Institute of Basic Medical Sciences, Chinese Academy of Medical Sciences, School of Basic Medicine Peking Union Medical College, Beijing 100005, China

**Keywords:** BRCA1, BRCA2, Epidermal growth factor receptor, Ovarian cancer

## Abstract

**Background:**

Both BRCA1 and epidermal growth factor receptor (EGFR) play a critical role in ovarian cancer progression. However, the crosstalk between BRCA1 and EGFR signaling pathways in ovarian cancer remains largely unknown.

**Methods:**

The effect of BRCA1 on EGFR was assessed in 146 serous ovarian cancer patients (28 pairs of BRCA1-mutated or not, 23 pairs of BRCA2-mutated or not, and 22 pairs with hypermethylated BRCA1 promoter or not). BRCA1 promoter methylation was analyzed by bisulfite sequencing using primers flanking the core promoter region. Expression levels of BRCA1 and EGFR were assessed by immunohistochemistry and real-time PCR. The knockdown and overexpression of BRCA1 were achieved using a lentiviral vector in 293 T cells, SKOV3 ovarian cancer cells, and primary non-mutated and BRCA1-mutated ovarian cancer cells.

**Results:**

EGFR expression was increased in all cancer tissues compared to normal tissues. Additionally, EGFR expression was higher in normal tissues of BRCA1-mutated patients, and was further increased in cancer tissues; EGFR levels were also significantly elevated in ovarian cancer with promoter hypermethylation-mediated inactivation of BRCA1. BRCA1 knockdown was an effective way to activate EGFR expression in ovarian cancer cells.

**Conclusions:**

These results indicate that BRCA1 may be a potential trigger in transcriptional regulation of EGFR in the development of ovarian cancer.

## Background

Ovarian cancer is characterized by a high rate of mortality among gynecologic oncology patients [1]. To date, although the exact cause of ovarian cancer remains largely unknown, BRCA mutations are known hereditary factors, and the risk of ovarian cancer conferred by BRCA mutations can be regulated by both genetic and environmental components [2]. The epidermal growth factor receptor (EGFR) is a member of the ErbB family of receptor tyrosine kinases that exert a direct effect on ovarian cell proliferation, migration, and invasion, as well as angiogenesis [3]. The overexpression of EGFR frequently occurs in ovarian cancer tissues [3,4] and correlates with poor prognosis of the patients [5,6]. Notably, emerging evidence has established that: (i) EGFR is a potential link between genetic and environmental interactions [7]; (ii) EGFR and BRCA1 can be found in the same protein complex, and convergence exists between EGFR- and BRCA1-related signaling pathways [8,9]; and (iii) BRCA1 mutations are vulnerable to the development of EGFR-positive cancers [10]. Therefore, insights into the complex interrelationship between BRCA and EGFR might improve our understanding of the basic molecular mechanism of ovarian cancer. For this reason, the present study was undertaken to investigate EGFR expression after BRCA inactivation events (mutation, promoter methylation, or knockdown), and to provide novel insights into the regulatory mechanism of EGFR.

## Methods

### Patients and tissue collection

This study was approved by the Institutional Review Board at China Medical University. Serous ovarian cancer patients (28 pairs of BRCA1-mutated or not, 23 pairs of BRCA2-mutated or not, and 22 pairs with hypermethylated BRCA1 promoter or not) were enrolled between 2010 and 2012, and all patients gave informed consent. Fresh tumor samples, adjacent normal ovarian tissues, ascites, and blood samples were obtained at the time of primary surgery before any chemotherapy or radiotherapy. Hematoxylin and eosin staining of the samples for histopathological diagnosis and grading were performed by three staff pathologists using the World Health Organization criteria. All patients were screened for BRCA1 and 2 mutations by multiplex polymerase chain reaction (PCR) with complete sequence analysis, as previously reported [11]. Their characteristics are given in Additional file 1.

### Cell culture and lentiviral transfection

Primary ovarian cancer cells were obtained from the ascites of patients undergoing surgery for ovarian cancer and cultured in RPMI 1640 with 10% fetal bovine serum (Invitrogen, CA, USA) as described previously [12]. Human 293 T cells and SKOV3 ovarian cancer cells were maintained in DMEM with 10% fetal bovine serum (Invitrogen). Lentiviral vectors expressing short hairpin RNAs (shRNAs) against BRCA1 (NM_007299) were obtained from Genechem Co., Ltd (Shanghai, China), and synthesized as follows: forward, 5′-CCGGAACCTGTCTCCACAAAGTGTGCTCGAGCACACTTTGTGGAGACAGGTTTTTTTG-3′, and reverse, 5′-AATTCAAAAAAACCTGTCTCCACAAAGTGTGCTCGAGCACACTTTGTGGAGACAGGTT-3′. The non-silencing shRNA sequence was used as a negative control and synthesized as follows: forward, 5′-ccggTTCTCCGAACGTGTCACGTctcgagACGTGACACGTTCGGAGAAtttttg-3′, and reverse, 5′-aattcaaaaaTTCTCCGAACGTGTCACGTctcgagACGTGACACGTTCGGAGAA-3′. For overexpression of BRCA1, the open reading frame of BRCA1 (NM_007299) was cloned into the lentiviral vector GV287 (Ubi-MCS-3FLAG-SV40-EGFP) (Genechem). Transfections were performed using polybrene and enhanced infection solution (Genechem) according to the manufacturer’s recommended protocol.

### Real-time PCR and immunohistochemical analysis

Real-time PCR and immunohistochemistry were performed as previously described [11]. The specific primer sequences for real-time PCR were as follows: EGFR, 5′- GCGAATTCCTTTGGAAAACC-3′ (F) and 5′- AAGGCATAGGAATTTTCGTAGTACA-3′ (R); BRCA1, 5′-GGCTATCCTCTCAGAGTGACATTT-3′ (F) and 5′-GCTTTATCAGGTTATGTTGCATGG-3′ (R); GAPDH, 5′-AGGTGAAGGTCGGAGTCA-3′ (F) and 5′-GGTCATTGATGGCAACAA-3′(R). The primary antibody for immunohistochemistry was rabbit anti-EGFR of human origin (1:250; Santa Cruz Biotechnology, CA, USA). Immunostaining was evaluated by two independent pathologists, blinded to the identity of subject groups. Area quantification was performed with a light microscope at a magnification of 400× and analyzed by Image-Pro Plus 6.0 (Media 2 Cybernetics, USA). The intensity of staining was divided into 10 units.

### Bisulfite sequencing

Genomic DNA extracted from ovarian cancer and normal ovarian tissue with a TIANamp Genomic DNA kit (Tiangen Biotech, Beijing, China) was subjected to bisulfite conversion using the EZ DNA Methylation-Direct kit (Zymo Research, Orange, USA) following the manufacturer’s instructions. The conversion efficiency was estimated to be at least 99.6%. The DNA was then amplified by nested PCR. After gel purification, cloning, and transformation into *Escherichia coli* Competent Cells JM109 (Takara, Tokyo, Japan), 10 positive clones of each sample were sequenced to ascertain the methylation patterns of each CpG locus. The following primers were used: round I, 5′-TTGTAGTTTTTTTAAAGAGT-3′ (F) and 5′-TACTACCTTTACCCAAAACAAAA-3′ (R); and round II, 5′-GTAGTTTTTTTAAAGAGTTGTA-3′ (F) and 5′-ACCTTTACCCAAAACAAAAA-3′ (R). The conditions were as follows: 95°C for 2 min, 40 cycles of 30 s at 95°C, 30 s at 56°C, and 45 s at 72°C, then 72°C for 7 min.

### Statistical analysis

The data are presented as mean ± standard deviation (SD). Statistical differences in the data were evaluated by a Student’s *t*-test or one-way analysis of variance (ANOVA) as appropriate, and were considered significant at *P* < 0.05.

## Results

### Differences in expression patterns of EGFR in non-mutated and BRCA1- or BRCA2-mutated ovarian cancer

**Figure 1:**
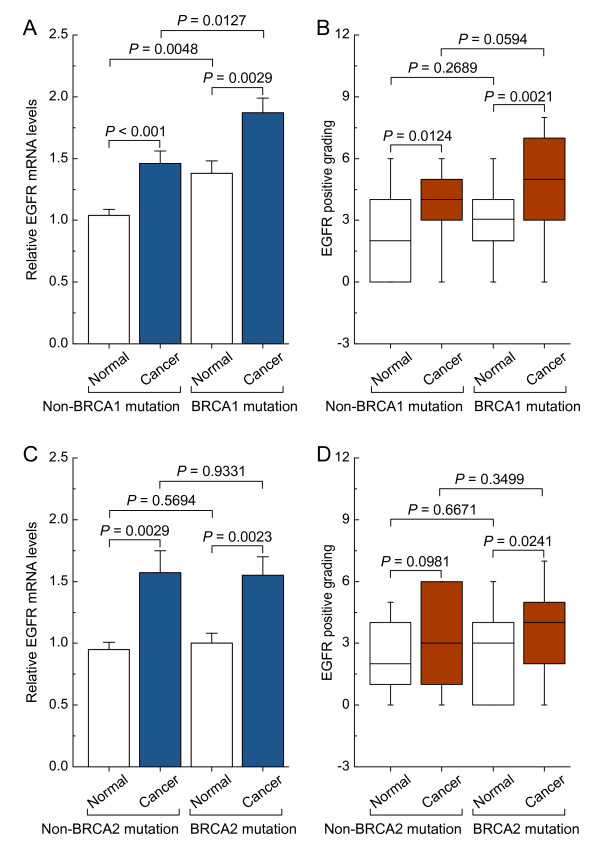
**EGFR expression patterns in non-mutated and BRCA1- or BRCA2-mutated ovarian cancer. A** and **C**, relative EGFR mRNA levels were measured in non-mutated and BRCA1- or BRCA2-mutated ovarian cancer, and their adjacent normal tissue. Bar graphs show mean ± SD. **B** and **D**, EGFR protein levels assessed by immunohistochemistry in non-mutated and BRCA1- or BRCA2-mutated ovarian cancer, and their adjacent normal tissue. The intensity of staining was divided into 10 units.

Real-time PCR and immunohistochemical analysis showed that the levels of EGFR mRNA and protein were increased in non-mutated and BRCA1-mutated ovarian cancer compared with their adjacent normal tissue. It is interesting to note that BRCA1-mutated ovarian cancer showed dramatically increased expression of EGFR compared with the remaining three groups (Figure 1A and B). However, although the levels of EGFR mRNA and protein were increased in non-mutated and BRCA2-mutated ovarian cancer compared with their adjacent normal tissue, there was no significant difference in the expression of EGFR between the non-mutated and BRCA2-mutated groups, including ovarian cancer and normal ovarian tissue (Figure 1C and D).

### Reduced expression of BRCA1 mediated by BRCA1 promoter hypermethylation is inversely correlated with EGFR levels

**Figure 2:**
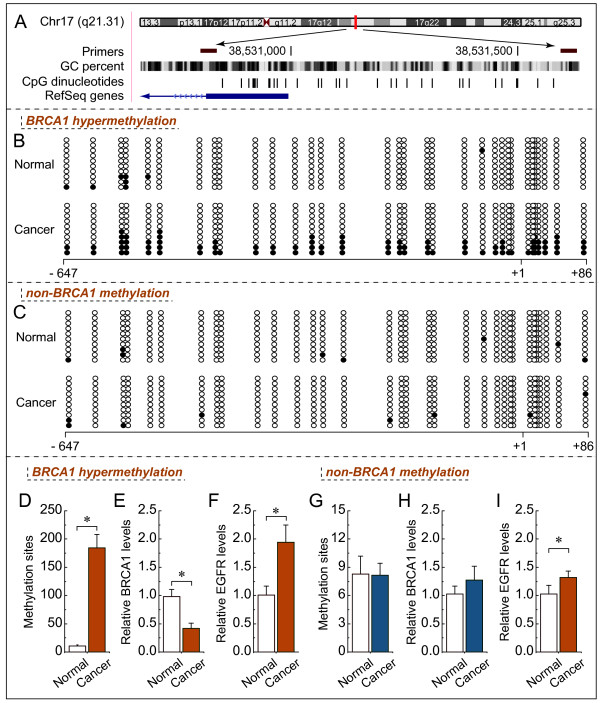
**EGFR expression patterns in ovarian cancer with hypermethylated promoter-mediated BRCA1 inactivation. A**, the location of CpG sites in the core promoter region of the BRCA1. Genomic coordinates are shown, along with the primer-amplified fragments, GC percentage, location of individual CpG dinucleotides (dashes), and BRCA1 RefSeq gene (exon 1 is shown as a blue box and the intron is shown as an arrowed line). The arrow indicates the direction of transcription. **B** and **C**, comparative analysis of methylation patterns in the core promoter region of BRCA1 in ovarian cancer and adjacent normal tissue. The circles correspond to the CpG sites denoted by black dashes in **A**. Closed circles, methylation; open circles, unmethylated. Ten individual clones were sequenced for each sample. **D** and **G**, summary of the methylation levels of BRCA1 core promoter from the measurements shown in **B** and **C**, respectively. **E** and **H**, relative BRCA1 mRNA levels were measured in ovarian cancer with identified hypermethylated or unmethylated BRCA1 promoter, compared with their adjacent normal tissue. **F** and **I**, relative EGFR mRNA levels were measured in ovarian cancer with identified BRCA1 inactivation or not, respectively. Bar graphs show mean ± SD. * *P* < 0.05 *vs*. normal.

In mammals, promoter methylation is an epigenetic modification involved in regulating gene expression [13]. Consistent with this idea, we showed that ovarian cancer tissue with a hypermethylated BRCA1 promoter (Figure 2B and D*, P* < 0.05) displayed reduced expression of BRCA1 (Figure 2E*, P* < 0.05) compared with adjacent normal tissue. However, no significant BRCA1 expression differences (Figure 2H*, P* > 0.05) were observed in ovarian cancer with an unmethylated BRCA1 promoter (Figure 2C and G*, P* > 0.05) compared with adjacent normal tissue. Based on these considerations, the low levels of BRCA1 mediated by promoter hypermethylation was an appropriate model for investigating the physiological relationship between BRCA1 and EGFR. Notably, the expression levels of EGFR were markedly increased (Figure 2F*, P* < 0.05), along with a hypermethylated promoter-mediated BRCA1 deficiency in ovarian cancer (Figure 2E*, P* < 0.05). However, although the expression of EGFR was also increased in ovarian cancer tissue (Figure 2I*, P* < 0.05) along with no significant difference in BRCA1 promoter methylation or expression (Figure 2G and H*, P* > 0.05), the increased levels of EGFR was not significant compared with ovarian cancer with BRCA1 deficiency.

### BRCA1 can regulate EGFR expression in ovarian cancer cells

**Figure 3:**
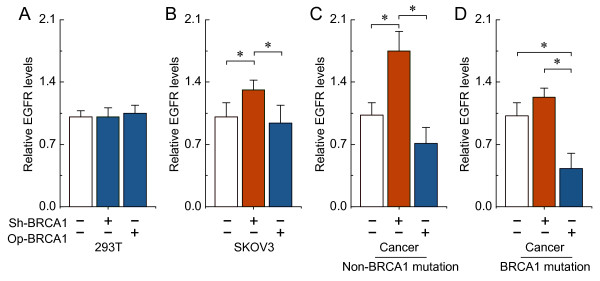
**Effects of BRCA1 on EGFR expression. A–D**, relative EGFR mRNA levels after the overexpression or knockdown of BRCA1 in 293 T cells, human SKOV3 ovarian cancer cells, and primary non-mutated and BRCA1-mutated ovarian cancer cells. Bar graphs show mean ± SD. * *P* < 0.05 *vs*. normal. Sh, short hairpin RNAs; Op, overexpression.

To further confirm the role of BRCA1 in the regulation of EGFR, the effects of overexpression or knockdown of BRCA1 were evaluated in 293 T cells, human ovarian cancer cell line SKOV3, and primary ovarian cancer cells with identified BRCA1 mutations or no BRCA1 mutations. The results indicated that there were no significant changes in the expression of EGFR after the overexpression or knockdown of BRCA1 in 293 T cells (Figure 3A). Interestingly, we observed that the knockdown of BRCA1 was an effective way to induce an increase of EGFR levels in SKOV3 and non-BRCA1-mutated ovarian cancer cells (Figure 3B and C). In addition, the overexpression of BRCA1 can effectively reduce the expression of EGFR in BRCA1-mutated ovarian cancer cells (Figure 3D).

## Discussion

In this study, we report an association between BRCA1 and EGFR status in ovarian cancer cells: (i) although EGFR expression was increased in BRCA1- and BRCA2-mutated ovarian cancer, only the BRCA1-mutated group exhibited dramatically increased expression of EGFR compared with the non-BRCA1-mutated group; (ii) BRCA1 inactivation (BRCA1 mutation or promoter hypermethylation) dramatically increased the expression of EGFR; and (iii) BRCA1 knockdown was an effective way to activate the EGFR gene. These results suggest that BRCA1 may be a potential regulator of EGFR in ovarian cancer, although a similar phenomenon has even been observed in breast cancer [14]. It appears that BRCA1 rather than BRCA2 may be a potential regulator of EGFR expression. In agreement with these findings, Nisman suggested that the concentration of soluble EGFR was significantly higher in women with BRCA1 mutations than in controls and women with BRCA2 mutations [8]. Interestingly, the activation effect due to the loss of BRCA1 was primarily observed in cells originating from ovarian cancer, while 293 T cells were insensitive to the overexpression or knockdown of BRCA1. Hence, the induced expression of EGFR was likely to be the result of a complex interaction of special factors in ovarian cancer cells. Notably, several studies suggest that BRCA1 haploinsufficiency is more likely to become cancerous compared with the non-BRCA1-mutated group, due to an extraordinary ability for clonal growth and proliferation [15]. EGFR also plays an important role in regulating cell proliferation and resistance to cell apoptosis during cancer development [3]. As shown in Additional file 2 (methods shown in Additional file 3), BRCA1 knockdown-mediated EGFR overexpression is associated with increased proliferation, and proliferative effects were reversed by the EGFR inhibitor erlotinib. Moreover, patients with low BRCA1-related high levels of EGFR showed a trend for poor survival (Additional file 4, methods shown in Additional file 3). Therefore, it can be predicted that BRCA1 inactivation-related high levels of EGFR may be involved in promoting ovarian cancer progression. To date, it is not fully understood how BRCA1 represses EGFR gene expression at the molecular level. However, is it possible that the repression takes place at the transcriptional level? Some insight was gained by a study demonstrating that BRCA1 is an important transcriptional regulator, which modulates the translational efficiency of approximately 7% of the mRNAs expressed in human breast cancer cell line MCF-7 [16]. A growing body of evidence suggests that BRCA1 has extensive cellular effects on hormone receptor signaling pathways. For example, BRCA1 can inhibit progesterone receptor (PR) activity in the PR-positive human breast cancer cell line T47D [17,18] and repress estrogen receptor-alpha activity in MCF-7 cells [19]. BRCA1 may also be a potential regulator of the insulin-like growth factor 1 receptor in human breast cancer cell line HCC1937 [20]. However, to date, there have been few reports about the interactions between BRCA1 and EGFR in ovarian cancer.

## Conclusions

The present study supports the theory that the EGFR gene is also a physiologically relevant downstream target for BRCA1. The data presented in this study emphasize the convergence of the EGFR-mediated cell proliferation pathway and BRCA1-mediated antitumor mechanism. Clarifying the complex interactions between BRCA1 and EGFR signaling pathways at the transcriptional, posttranscriptional, and epigenetic levels may improve our understanding of the basic molecular mechanism of ovarian cancer.

## Abbreviations

ANOVA: Analysis of variance; EGFR: Epidermal growth factor receptor; PCR: Polymerase chain reaction; PR: Progesterone receptor; shRNAs: Short hairpin RNAs; SD: Standard deviation,

## Competing interests

The authors declare that they have no competing interests.

## Authors’ contributions

DL and QY conceived of the study, participated in its design and drafted the manuscript. DL, FFB and JMC carried out data acquisition and interpretation. CC and CYL participated in the design of the study and performed the statistical analysis. All authors read and approved the final manuscript.

## Supplementary Material

Additional file 1: Table S1 Clinical characteristics for the 28 BRCA1-mutated serous ovarian cancer patients. **Table S2**: Clinical characteristics for the 23 BRCA2-mutated serous ovarian cancer patients. Click here for file

Additional file 2 Cell proliferation after the overexpression of BRCA1, or knockdown of BRCA1 plus erlotinib or not. Click here for file

Additional file 3 Supplementary methods. Click here for file

Additional file 4 Univariate analysis of overall survival for ovarian cancer patients with low BRCA1-high EGFR expression and high BRCA1-low EGFR expression. Click here for file
