# Supplementary figures and images for: Effect of BRCA1 on epidermal growth factor receptor in ovarian cancer
Source: J Exp Clin Cancer Res. 2013 Dec 9;32(1):102. doi: 10.1186/1756-9966-32-102 (PMC4029425; doi:10.1186/1756-9966-32-102)

## Additional file 2

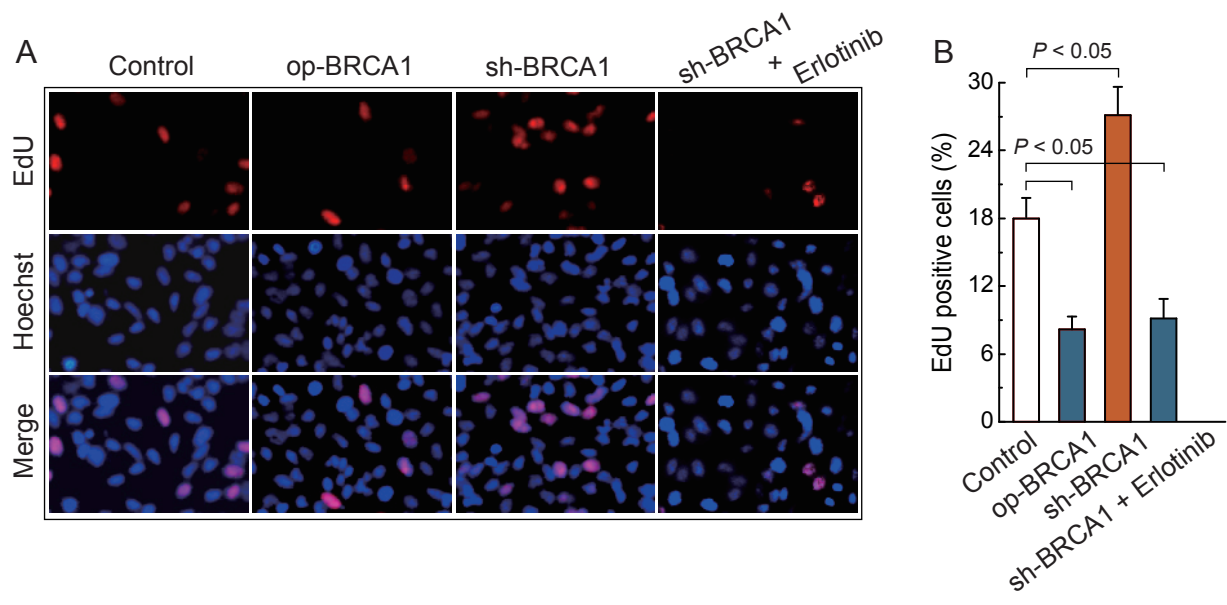

Supplement: Additional file 2 — Cell proliferation after the overexpression of BRCA1, or knockdown of BRCA1 plus erlotinib or not. [file 1756-9966-32-102-S2.pdf]

Additional file 4

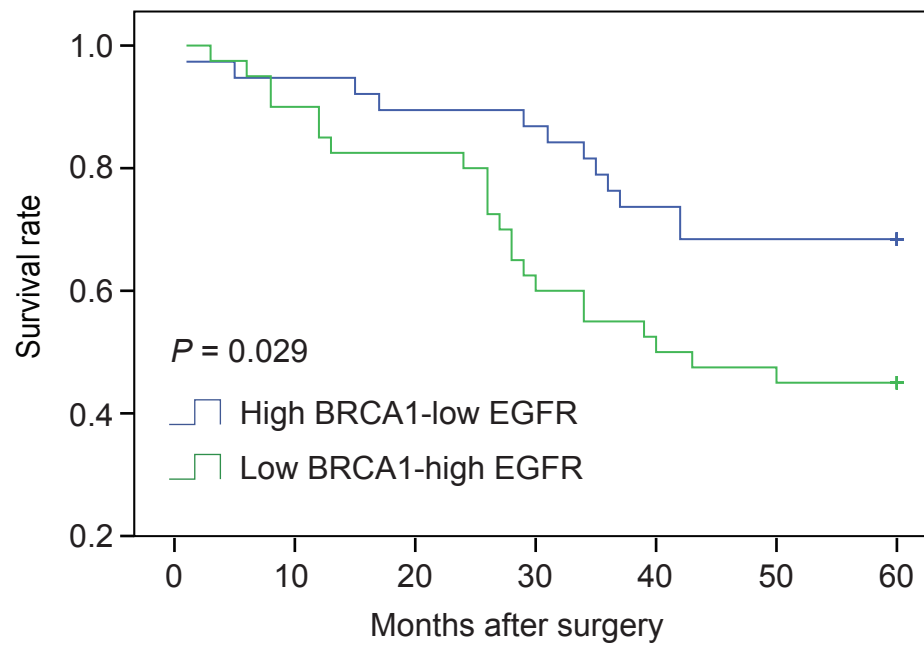

Supplement: Additional file 4 — Univariate analysis of overall survival for ovarian cancer patients with low BRCA1-high EGFR expression and high BRCA1-low EGFR expression. [file 1756-9966-32-102-S4.pdf]
