# Supplementary material for: Effect of BRCA1 on epidermal growth factor receptor in ovarian cancer
Source: J Exp Clin Cancer Res. 2013 Dec 9;32(1):102. doi: 10.1186/1756-9966-32-102 (PMC4029425; doi:10.1186/1756-9966-32-102)
Supplement: Additional file 3 — Supplementary methods. [file 1756-9966-32-102-S3.pdf]

Supplementary Methods

Erlotinib treatment and cell proliferation assay

Erlotinib hydrochloride was purchased from Santa Cruz Biotechnology (CA, USA). Primary ovarian cancer cells were exposed to 1 μM erlotinib 48 h after BRCA1 knockdown. Cell proliferation was determined using the Cell-Light™ EdU Apollo®643 In Vitro Imaging Kit (Ribobio, Guangzhou, China) according to the manufacturer's instructions. The proliferation rate (EdU incorporation rate) was expressed as the ratio of EdU-positive cells to total Hoechst 33342-positive cells. Similar results were obtained in SKOV3 ovarian cancer cells. Blue indicates Hoechst 33342 labeling of cell nuclei; red indicates EdU labeling of nuclei of proliferative cells. sh: short hairpin RNAs; op: overexpression.

Univariate analysis of overall survival of patients with ovarian cancer

We performed a Kaplan-Meier analysis and log-rank tests for overall survival in defined prognostic subgroups: low BRCA1-high EGFR expression (n = 40) *vs*. high BRCA1-low EGFR expression (n = 38).
